# A standardized ECPR activation protocol and resuscitation efficiency, patient outcomes, and nursing cognitive load: a retrospective multicenter study

**DOI:** 10.3389/fcvm.2026.1899029

**Published:** 2026-10-07

**Authors:** Caiyan Qian, Minling Yang, Yinyin Wu, Liang Mei, Zhuoran Ma, Libing Ning, Peidong Yuan, Xiyang Li

**Affiliations:** 1 Cardiovascular Medicine Center, The Seventh Affiliated Hospital, Sun Yat-sen University, Shenzhen, Guangdong, China; 2 Cardiothoracic Surgery ICU, The First Affiliated Hospital, Sun Yat-sen University, Guangzhou, Guangdong, China; 3 ICU, Longgang Central Hospital of Shenzhen, Shenzhen, Guangdong, China; 4 Department of Ophthalmology, The Seventh Affiliated Hospital, Sun Yat-sen University, Shenzhen, Guangdong, China

**Keywords:** cognitive load, extracorporeal cardiopulmonary resuscitation, quality improvement, rapid response team, standardized activation protocol

## Abstract

**Objective:**

To evaluate changes in resuscitation efficiency, patient outcomes, and nursing cognitive load associated with implementation of a standardized extracorporeal cardiopulmonary resuscitation (ECPR) activation protocol.

**Methods:**

This retrospective multicenter observational study included 103 consecutive patients with in-hospital cardiac arrest who underwent ECPR at three tertiary hospitals in Guangdong Province, China. The control group included 30 patients treated from September 2019 to December 2023, and the post-implementation group included 73 patients treated from January 2024 to December 2025. Outcomes included resuscitation-process measures, clinical outcomes, and perceived nursing workload assessed using the NASA Task Load Index (NASA-TLX), a multidimensional measure of perceived workload.

**Results:**

Compared with the control group, the post-implementation group had shorter ECPR preparation time (2.87 ± 2.32 vs. 8.60 ± 1.34 min), ECMO priming time (14.83 ± 2.88 vs. 19.07 ± 1.18 min), and ECMO initiation time (35.01 ± 2.36 vs. 54.12 ± 3.55 min), and a higher one-time readiness rate of disposable supplies (100% vs. 50.0%; all *P* < 0.001). The total NASA-TLX score was lower after implementation (415 ± 26 vs. 452 ± 25, *P* < 0.001). Thirty-day survival was numerically higher in the post-implementation group but did not differ statistically (42.5% vs. 23.3%; crude OR 2.43, 95% CI 0.92–6.37; *P* = 0.069). Favorable neurological outcome at discharge was more frequent after implementation (30.1% vs. 10.0%; crude OR 3.88, 95% CI 1.07–14.15; *P* = 0.036), and the association persisted after adjustment for SAVE score and time-to-ECPR-decision (adjusted OR 3.79, 95% CI 1.02–14.08; *P* = 0.047).

**Conclusion:**

Implementation of the standardized ECPR activation protocol was associated with shorter ECPR preparation, priming, and initiation times, lower perceived nursing workload, and higher odds of favorable neurological outcome at discharge. Thirty-day survival was numerically higher but did not differ statistically. Given the retrospective pre-post design, these findings should be interpreted as associations rather than causal effects.

## 1 Introduction

Extracorporeal cardiopulmonary resuscitation (ECPR) refers to the use of veno-arterial extracorporeal membrane oxygenation (VA-ECMO) in selected patients with potentially reversible causes who do not achieve sustained return of spontaneous circulation during conventional cardiopulmonary resuscitation (1). Reported ECPR survival in China remains lower than rates described in some international series (2), and current consensus emphasizes timely evaluation and implementation in appropriately selected patients (3). ECPR is operationally complex and requires close coordination within a specialized team. Cognitive Load Theory (CLT) proposes that excessive demands on finite cognitive resources can impair task performance, providing a framework for workflow standardization in high-pressure clinical settings (4, 5). Standardized activation models may facilitate coordination and task execution (6). In January 2024, a standardized ECPR activation protocol was implemented across three tertiary hospitals as a multicenter clinical quality-improvement initiative. The present study was a retrospective multicenter observational evaluation of changes in resuscitation efficiency, patient outcomes, and nursing cognitive load associated with protocol implementation.

## 2 Materials and methods

### 2.1 General information

A retrospective multicenter study was conducted across three tertiary hospitals in Guangdong Province, China [The Seventh Affiliated Hospital, Sun Yat-sen University, The First Affiliated Hospital, Sun Yat-sen University, Longgang Central Hospital of Shenzhen]. Patients who underwent ECPR following in-hospital cardiac arrest (IHCA) at the participating centers between September 2019 and December 2025 were consecutively enrolled. A total of 103 patients were included and divided into a control group and a post-implementation group based on the treatment protocol received. The control group comprised 30 patients who received conventional ECPR between September 2019 and December 2023. The post-implementation group comprised 73 patients who underwent ECPR following the standardized ECPR activation protocol between January 2024 and December 2025. The inclusion criteria for ECPR patients are shown in Figure 1.

**Figure 1:**
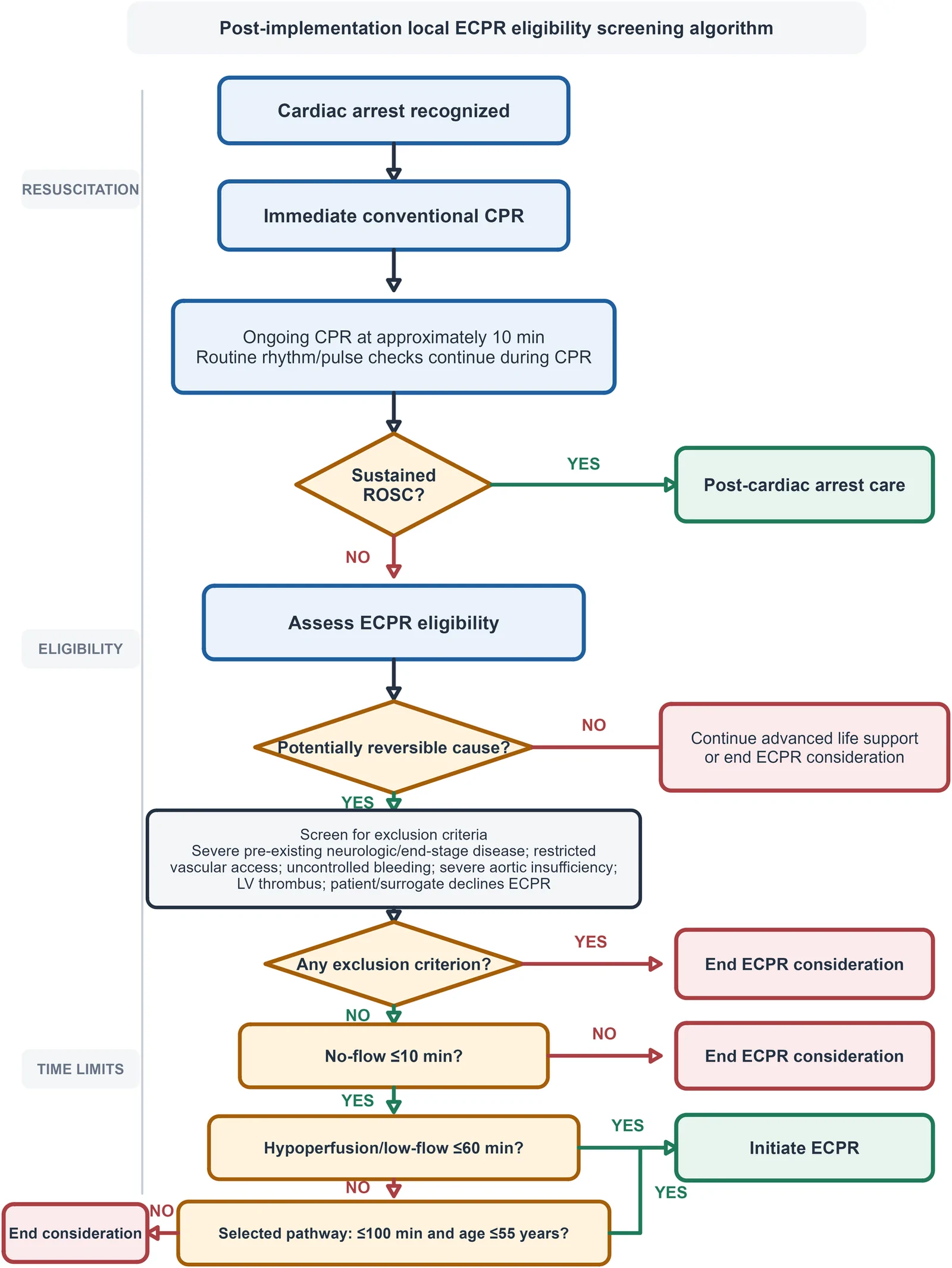
ECPR eligibility screening algorithm used in the post-implementation period. For historical controls, the same thresholds were applied retrospectively for study eligibility. CPR, cardiopulmonary resuscitation; ECPR, extracorporeal cardiopulmonary resuscitation; ROSC, return of spontaneous circulation.

A total of 35 core nursing staff members who participated in the ECPR resuscitation efforts across the three centers during the study period were enrolled as the survey subjects for cognitive load assessment. Inclusion criteria were: 1) holding a valid nurse practice certificate; 2) having worked in critical care for ≥3 years; 3) having directly participated in ECPR resuscitation at one of the participating centers; 4) volunteering to participate in this study and completing the questionnaire survey. Exclusion criteria were: 1) personnel who left or were transferred from their posts during the study period; 2) nursing staff in internship or advanced training; 3) those who failed to complete the questionnaire or withdrew from the study.

The study was approved by the Ethics Committee of The Seventh Affiliated Hospital, Sun Yat-sen University (Shenzhen) (approval no. KY-026-044-01). The two collaborating hospitals provided formal written institutional authorization, accepted the lead-center ethical review conclusion, and did not conduct separate full local ethics committee review. All analyzed data were de-identified. The ethics committee waived individual research informed consent for this retrospective observational study. Clinical procedural consent for ECPR treatment was obtained from a patient surrogate, next-of-kin, or legal surrogate according to routine clinical practice and was separate from research consent.

### 2.2 Methods

#### 2.2.1 Control group

Patients in the control group received conventional ECPR management according to each center's practice at the time. CPR was initiated immediately after recognition of cardiac arrest. No uniform mandatory 10-min time point for formal ECPR eligibility assessment was used across the centers; candidate evaluation generally occurred after approximately 10–20 min of CPR. Team notification was hierarchical, a dedicated 24-hour ECPR roster was not consistently available, and material preparation and circuit priming often began after individually recalled personnel arrived. Cannulation was generally performed by percutaneous puncture without routine ultrasound guidance.

During the control period, the thresholds shown in Figure 1 were applied retrospectively as study eligibility criteria rather than as a uniform clinical workflow. Beginning in January 2024, the algorithm was adopted across the three centers as the formal screening pathway.

#### 2.2.2 Post-implementation group

Patients in the post-implementation group received ECPR management under the standardized activation protocol introduced across all three centers in January 2024 as a multicenter clinical quality-improvement initiative. The ECPR eligibility screening algorithm is shown in Figure 1, and the coordinated implementation workflow is shown in Figure 2.

**Figure 2:**
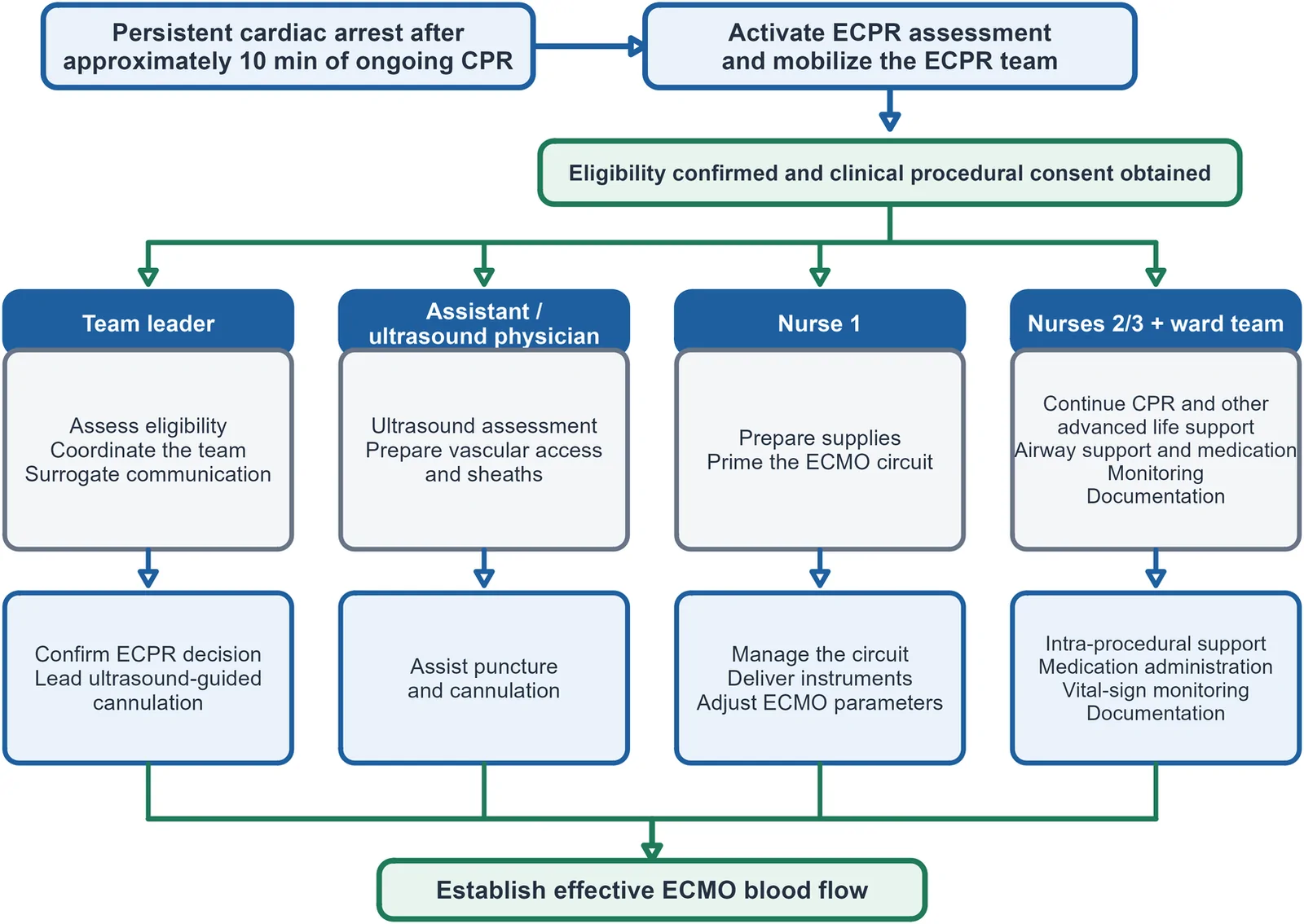
Coordinated ECPR implementation workflow in the post-implementation period. Persistent cardiac arrest after approximately 10 min of ongoing CPR triggered formal ECPR assessment and team mobilization rather than automatic ECPR initiation. Detailed task allocation is summarized in the figure.

Human Resource Management: A 24-hour on-call scheduling mechanism was established at each center. The ECPR team at each center consisted of at least four qualified physicians who participated in the rotation, with the physician on duty serving as the team leader responsible for ECPR decision-making. All participating physicians held attending physician titles or above and possessed standardized ECMO training certificates. The chief resident of the Department of Cardiac Surgery or equivalent served as the assistant. Two to three critical care nurses participated in the daily ECPR team rotation, with at least one nurse per shift holding a training certificate from a national ECMO training center. An on-call physician from the Department of Ultrasound or radiology was also integrated into the ECPR team rotation.

Standardized Material Management: At each center, an ECMO supply cart was configured, containing categorized puncture boxes, cannulation boxes, and priming boxes with corresponding item checklists. All consumables and the ECMO console were placed in fixed locations, with regular inspection and replenishment, achieving standardized configuration of resuscitation supplies.

Personnel Training: A unified training program was developed by the lead center and disseminated across all three sites. Specialist nurses organized theoretical and practical training for critical care nurses participating in the ECPR rotation, covering ECMO fundamentals, priming techniques, alarm management, and patient care during ECMO. Scenario-based drills were conducted, requiring each nurse to complete at least three full-process ECPR scenario simulations. Competency was defined as completing ECMO priming within 15 min and achieving a score of ≥80 on theoretical assessments. A physician puncture training program was developed in collaboration with the medical team, introducing ultrasound-guided puncture techniques. Physicians participating in the ECPR rotation and the cardiac surgery chief resident or equivalent were required to complete at least one puncture skills practice per month to maintain and enhance cannulation skills.

Protocol Standardization and Process Optimization: Through expert consultation, literature review, and multidisciplinary team discussions, a series of institutional protocols were developed by the lead center and subsequently adopted across all three sites, including: “ECPR Patient Management Standards,” “ECMO Implementation Standardized Procedure,” “ECMO Malfunction and Emergency Response Plan,” and the revised “ECPR Initiation Procedure”—totaling more than 10 protocol documents.

Role Delineation and Responsibilities: For persistent cardiac arrest after approximately 10 min of ongoing high-quality CPR, the primary ward activated formal ECPR eligibility assessment and mobilized the ECPR team. ECPR proceeded only after eligibility was confirmed and clinical procedural consent was obtained. Detailed role allocation and parallel task execution are shown in Figure 2.

### 2.3 Outcome measures

ECPR process outcomes included ECPR preparation time, one-time readiness rate of disposable items, ECMO priming time, and ECMO initiation time.

Time-to-ECPR-decision was recorded as a baseline time covariate and was defined as the interval from recognition of cardiac arrest and initiation of CPR to the clinical decision to activate ECPR.

ECMO initiation time was defined as the interval from the clinical ECPR activation decision to establishment of effective ECMO blood flow.

Nursing workload was assessed using the NASA Task Load Index (NASA-TLX), a multidimensional measure of perceived workload. The Chinese version has a reported Cronbach's *α* of 0.707 (7) and includes mental demand, physical demand, temporal demand, performance, effort, and frustration. Each dimension is scored from 0 to 100; performance is reverse scored. The total score ranges from 0 to 600, with higher scores indicating greater perceived workload.

Patient outcomes included successful ECMO weaning, 30-day survival, and Cerebral Performance Category (CPC) score at hospital discharge. CPC 1–2 was classified as a favorable neurological outcome.

### 2.4 Data collection

Patient-related data were obtained from the electronic medical record and paper medical record systems of the participating centers. For the cognitive load assessment, the same cohort of 35 core nursing staff (as detailed in Section 2.1) who actively participated in ECPR resuscitations during both the control and post-implementation periods were enrolled as the paired survey subjects.

Following each ECPR resuscitation, participants completed the Chinese NASA-TLX questionnaire within 24 h and were instructed to evaluate only the workload associated with that specific resuscitation event. The questionnaire was completed independently, without discussion with other team members, using identical standardized instructions and procedures in both periods.

### 2.5 Statistical analysis

SPSS 22.0 was used for statistical analysis. Normally distributed continuous patient variables were summarized as mean ± SD and compared between groups using independent-samples t tests. Pre- and post-implementation NASA-TLX scores from the same 35 nurses were compared using paired-samples t tests. Categorical variables were summarized as counts and percentages and compared using *χ*^2^ tests or Fisher's exact tests when any expected cell count was <5. Two-sided *P* < 0.05 was considered statistically significant. Baseline severity was described using the SAVE (Survival After Veno-arterial ECMO) score. Time-to-ECPR-decision was defined as the interval from recognition of cardiac arrest and initiation of CPR to the clinical decision to activate ECPR; ECMO initiation time was defined as the interval from the clinical ECPR activation decision to establishment of effective ECMO blood flow. Multivariable logistic regression models for clinical outcomes included study group, SAVE score, and time-to-ECPR-decision as covariates. Because of the limited number of outcome events, no additional covariates were added. Results are reported as adjusted odds ratios (ORs) with 95% confidence intervals (CIs).

## 3 Results

### 3.1 General information of study subjects

A total of 103 patients with IHCA who underwent ECPR were included: 30 in the control group and 73 in the post-implementation group. No statistically significant between-group differences were observed in the reported baseline characteristics, including age, sex, causes of cardiac arrest, SAVE score, or time-to-ECPR-decision (Table 1).

**Table 1:** Comparison of basic information of two groups of patients (*n* = 103).

Characteristic	Control group (*n* = 30)	Post-implementation group (*n* = 73)	*t*/*χ*^2^ value	*P* value
Age	53 ± 14	53 ± 16	−0.047	0.962
Gender			0.419	0.518
Male	22 (73.3%)	47 (64.4%)		
Female	8 (26.7%)	26 (35.6%)		
Causes of cardiac arrest				
Cardiogenic shock	10 (33.3%)	29 (39.7%)	0.362	0.547
Acute myocardial infarction	5 (16.7%)	14 (19.2%)	0.087	0.768
Infection	6 (20.0%)	6 (8.2%)	—	0.104^a^
Malignant arrhythmia	2 (6.7%)	9 (12.3%)	—	0.502^a^
Myocarditis	2 (6.7%)	7 (9.6%)	—	0.723^a^
Other	5 (16.7%)	8 (11.0%)	—	0.513^a^
SAVE score	−0.87 ± 3.06	0.27 ± 3.18	−1.672	0.098
Time-to-ECPR-decision (min)	20.10 ± 5.93	20.90 ± 5.03	−0.700	0.486

Other includes trauma, burns, hemorrhagic shock, electric shock, drowning, poisoning, and pulmonary embolism.

All enrolled patients in this study were in-hospital cardiac arrest (IHCA) cases. Age values are rounded to the nearest whole year for display; the t statistic and *P* value are unchanged.

SAVE, Survival After Veno-arterial ECMO score.

a Fisher's exact test.

A total of 35 core nursing staff members who participated in all ECPR resuscitation efforts were selected as the survey subjects for cognitive load assessment. Among them, 21 were female (60.0%) and 14 were male (40.0%); the mean age was 32.5 ± 5.7 years; the mean years of work experience in intensive care was 9.6 ± 5.4 years; the distribution of nursing levels was: 14 at N2 level (40.0%), 17 at N3 level (48.6%), and 4 at N4 level (11.4%); all nursing staff held a bachelor's degree; 13 nursing staff members (37.1%) had received training at a national ECMO training center, among whom 2 (5.7%) were certified as provincial-level ECMO specialist nurses in Guangdong. It is noteworthy that this identical cohort of 35 nurses completed the NASA-TLX assessment for both the pre-implementation (control period) and post-implementation phases, serving as their own controls for the cognitive load comparison.

### 3.2 Comparison of ECPR initiation timeliness indicators between the two patient groups

Compared with the control group, the post-implementation group showed significantly shorter ECPR preparation time (2.87 ± 2.32 vs. 8.60 ± 1.34 min, *P* < 0.001), ECMO priming time (14.83 ± 2.88 vs. 19.07 ± 1.18 min, *P* < 0.001), and ECMO initiation time (35.01 ± 2.36 vs. 54.12 ± 3.55 min, *P* < 0.001). The one-time readiness rate of disposable items increased from 50.0% to 100% (*P* < 0.001) (Table 2).

**Table 2:** Comparison of ECPR initiation timeliness indicators between the two groups (*n* = 103).

Indicator	Control group (*n* = 30)	Post-implementation group (*n* = 73)	Difference^a^	*P* value
Preparation time (min)	8.60 ± 1.34	2.87 ± 2.32	−5.73 min	<0.001
Priming time (min)	19.07 ± 1.18	14.83 ± 2.88	−4.24 min	<0.001
ECMO initiation time (min)	54.12 ± 3.55	35.01 ± 2.36	−19.11 min	<0.001
One-time readiness rate of items (%)	50.00%	100.00%	+50.0 pp	<0.001

Difference = post-implementation minus control; time differences are in minutes and the readiness-rate difference is in percentage points.

### 3.3 Comparison of ECMO weaning success rate, 30-day survival rate, and favorable neurological function rate at discharge between the two groups

Clinical outcomes are shown in Table 3. The 30-day survival proportion was numerically higher in the post-implementation group than in the control group, although the difference was not statistically significant (42.5% [31/73] vs. 23.3% [7/30]; crude OR 2.43, 95% CI 0.92–6.37; *P* = 0.069). Favorable neurological outcome at discharge was more frequent in the post-implementation group (30.1% [22/73] vs. 10.0% [3/30]; crude OR 3.88, 95% CI 1.07–14.15; *P* = 0.036).

**Table 3:** Comparison of 30-day survival rate, ECMO weaning success rate, and favorable neurological function rate at discharge between the two groups (*n* = 103).

Outcome	Control group (*n* = 30)	Post-implementation group (*n* = 73)	*χ*^2^ value	*P* value	Crude OR (95% CI)
ECMO weaning success rate	7/30 (23.3%)	31/73 (42.5%)	3.305	0.069	2.43 (0.92–6.37)
30-day survival rate	7/30 (23.3%)	31/73 (42.5%)	3.305	0.069	2.43 (0.92–6.37)
Favorable neurological outcome rate (at discharge)	3/30 (10.0%)	22/73 (30.1%)	4.388	0.036	3.88 (1.07–14.15)

### 3.4 Multivariable analysis of clinical outcomes

Multivariable logistic regression models adjusted for SAVE score and time-to-ECPR-decision are shown in Table 4. For 30-day survival, the adjusted OR was 2.62 (95% CI 0.97–7.09; *P* = 0.057), and the association was not statistically significant. Implementation of the standardized protocol was associated with higher odds of favorable neurological outcome at discharge, and this association persisted after adjustment (adjusted OR 3.79, 95% CI 1.02–14.08; *P* = 0.047). The Hosmer–Lemeshow test indicated no evidence of poor model fit (*P* > 0.05).

**Table 4:** Multivariable logistic regression analysis of 30-day survival and favorable neurological outcome.

Outcome variable	Variable	Adjusted OR	95% CI	*P* value
30-day survival	Standardized Protocol (vs. Control)	2.62	0.97–7.09	0.057
	SAVE Score (per 1-point increase)	0.99	0.87–1.13	0.845
	Time-to-ECPR-decision (per 1-min increase)	0.95	0.87–1.03	0.191
Favorable neurological outcome	Standardized Protocol (vs. Control)	3.79	1.02–14.08	0.047
	SAVE Score (per 1-point increase)	1.08	0.93–1.25	0.308
	Time-to-ECPR-decision (per 1-min increase)	0.96	0.87–1.05	0.344

All models were adjusted for SAVE score and time-to-ECPR-decision. An OR > 1 indicates a higher likelihood of the favorable outcome (30-day survival or favorable neurological outcome) in the post-implementation group compared with the control group. Hosmer–Lemeshow test *P* > 0.05 for both models, indicating good fit.

### 3.5 Comparison of nursing staff NASA-TLX scores before and after protocol implementation

Among the paired cohort of 35 nurses, the total NASA-TLX score was lower after implementation (415 ± 26 vs. 452 ± 25; *P* < 0.001). Mental demand, temporal demand, performance, effort, and frustration scores were also lower (all *P* < 0.001), whereas physical demand did not differ statistically (*P* = 0.173) (Table 5).

**Table 5:** Comparison of NASA-TLX dimension scores among nursing staff before and after protocol implementation (*n* = 35).

Dimension	Pre-implementation, mean ± SD	Post-implementation, mean ± SD	Paired *t* value	*P* value
Mental demand	84 ± 7	77 ± 7	−13.724	<0.001
Physical demand	78 ± 9	77 ± 9	−1.391	0.173
Temporal demand	86 ± 8	79 ± 9	−15.871	<0.001
Performance	29 ± 5	20 ± 6	−12.332	<0.001
Effort	90 ± 6	83 ± 7	−15.059	<0.001
Frustration level	85 ± 7	78 ± 8	−14.048	<0.001
Total score	452 ± 25	415 ± 26	−28.831	<0.001

Performance scores were reverse scored (100 - original score). Values are rounded to the nearest whole number for display; t statistics and *P* values are unchanged. *t* values are from paired-samples t tests.

## 4 Discussion

Following implementation of the standardized ECPR activation protocol, shorter ECPR preparation, priming, and ECMO initiation times and lower perceived nursing workload were observed across the three centers. The 30-day survival proportion was numerically higher but did not differ statistically. Implementation was associated with higher odds of favorable neurological outcome at discharge, and this association persisted after adjustment for SAVE score and time-to-ECPR-decision. These findings should be interpreted in the context of a retrospective pre-post comparison, in which learning effects, secular changes, and other unmeasured factors may also explain part of the observed differences.

Rapid ECPR implementation depends on coordinated patient selection, team activation, material preparation, circuit priming, and cannulation (1, 8–12). Reports from China and other countries describe ECMO responsibilities increasingly being assumed by intensive care or emergency teams (13, 14), underscoring the importance of reliable multidisciplinary workflows and nursing coordination (15–17). In this study, the standardized approach combined fixed supply locations, an on-call roster, predefined roles, ultrasound-guided cannulation, and scenario-based training. These components may have facilitated parallel task execution and reduced avoidable process delays. The observed associations with process efficiency and neurological outcome support further evaluation of this standardized approach rather than establishing its causal effectiveness.

The lower NASA-TLX scores observed after implementation were not accompanied by a statistically detectable change in physical demand. From a CLT perspective, fixed supplies and checklists may have reduced extraneous load; predefined roles and ultrasound guidance may have reduced demands arising from task complexity; and repeated scenario-based training may have supported development of team mental models (5, 18, 19). These mechanisms were not measured separately, however, and should be considered possible explanations rather than demonstrated mediators. The unchanged physical-demand score also suggests that the observed workload difference primarily concerned perceived cognitive and temporal demands.

For critical care team development, the findings are consistent with the potential value of combining technical training with workflow rehearsal, material standardization, and explicit task allocation. Specialist nurses may contribute to these processes through supply management, education, coordination, and monitoring of perceived workload (6, 20–24). Prospective evaluations are needed to determine whether the observed associations are reproducible and which protocol components are most important.

## 5 Strengths

This study provides experience from three tertiary-care settings and integrates operational, patient, and nursing outcomes. The paired NASA-TLX assessment adds a team-workload perspective, and the protocol description allows its workflow components to be examined in future studies.

## 6 Limitations

This retrospective pre-post study is subject to temporal bias, learning-curve effects, and secular changes in ECPR practice. Historical practices differed across centers, and group sizes were unequal. The NASA-TLX is subjective and may be affected by recall and response bias. The limited number of clinical events restricted multivariable adjustment, and residual or unmeasured confounding remains possible. Reported baseline characteristics did not differ statistically, but this does not establish equivalence between groups. These limitations preclude causal inference. Prospective multicenter studies are needed to assess whether the associations are reproducible and to better determine the effects of standardized ECPR activation workflows.

## 7 Conclusion

Implementation of the standardized ECPR activation protocol was associated with shorter ECPR preparation, priming, and ECMO initiation times, higher one-time supply readiness, and lower perceived nursing workload. Implementation was also associated with higher odds of favorable neurological outcome after adjustment for SAVE score and time-to-ECPR-decision. Thirty-day survival was numerically higher but did not differ statistically. Because the retrospective pre-post design precludes causal inference, these findings support further prospective evaluation of standardized ECPR activation workflows.

## Data Availability

The original contributions presented in the study are included in the article/Supplementary Material, further inquiries can be directed to the corresponding authors.
